# Survival effects of primary and metastatic surgical treatment in metastatic small intestinal tumors: A propensity score–matching study

**DOI:** 10.1371/journal.pone.0270608

**Published:** 2022-06-24

**Authors:** Zhongyi Zhou, Heming Ge, Yuqiang Li, Dan Wang, Cenap Güngör

**Affiliations:** 1 Department of General Surgery, Xiangya Hospital, Central South University, Changsha, China; 2 National Clinical Research Center for Geriatric Disorders, Xiangya Hospital, Central South University, Changsha, China; 3 Department of General Visceral and Thoracic Surgery, University Medical Center Hamburg-Eppendorf, Hamburg, Germany; Universitá Sapienza di Roma, ITALY

## Abstract

**Objective:**

To analyze the effects of primary tumor resection and metastatic lesion resection on the survival of metastatic small intestinal tumors.

**Methods:**

The research subjects were patients with metastatic small bowel tumors identified from 2004 to 2016 in the Surveillance, Epidemiology, and End Results (SEER) database. Propensity score matching and Kaplan–Meier analyses were performed to analyze the effect of surgery on the prognosis.

**Results:**

A total of 4,034 patients from the SEER database were analyzed. Both before and after the propensity score–matching analysis, the prognosis of patients who underwent primary tumor surgery and metastatic surgery was better than that of patients who did not undergo surgery; all were patients with metastatic small bowel adenocarcinoma (mSIA) or metastatic small intestinal neuroendocrine tumors (mSI-NETs) (all *p* < .005). Patients with mSIA and adequate lymph node dissection had a longer prognosis than mSIA patients with inadequate lymph node dissection, but this survival benefit was not present in mSI-NET patients. It made no difference in the prognosis of mSIA and mSI-NETs whether localized surgery or intestine-ectomy was performed. Patients with mSIA who underwent primary and metastatic excision plus chemotherapy had the best overall survival and cancer-specific survival rates, whereas mSI-NET patients who underwent primary and metastatic excision had the best overall survival and cancer-specific survival rates (all *p* < .001).

**Conclusion:**

In these carefully selected patients, primary tumor resection and/or metastatic lesion resection significantly improved the survival rates for patients with mSIA and mSI-NETs. The mSIA patients with resectable primary tumors seemed to require a sufficient number of lymph node dissections more than the patients with well-differentiated mSI-NETs.

## Introduction

Small bowel cancer is a rare neoplasm that accounts for only 3% of gastrointestinal cancers [1]. The overall frequency of gastrointestinal tumors has decreased, but the incidence of cancers of the small intestine has been rising by an average of 1.8% annually in the past decades [2]. An important aspect of the disease is that patients may have nonspecific symptoms and associated diagnostic delays, and this means that 30% to 32% of small bowel cancer patients have metastases at the time of diagnosis [3]; this is the main reason for the poor prognosis of small bowel cancer.

Adenocarcinoma and neuroendocrine tumors (NETs) are the most common histologic types of small intestinal cancers [2]. The therapeutic mainstay for metastatic small intestinal adenocarcinoma (mSIA) is systemic therapy, according to the National Comprehensive Cancer Network (NCCN) guidelines [2]. Previous studies confirmed the irreplaceable role of chemotherapy for mSIA [4, 5]. Primary tumor resection is mostly performed for mSIA with tumor-associated adverse events, which, in fact, are likely to occur during systemic chemotherapy [5]. Research exploring the efficacy of metastatic resection is scarce in mSIA patients. The removal of primary tumors and metastatic lesions is actually controversial in the setting of mSIA [6].

In patients with metastatic small intestinal NETs (mSI-NETs), the quality of life is seriously affected by both the carcinoid syndrome, which causes systemic manifestations, and the primary tumor, which causes local complications. Mesenteric and retroperitoneal fibrosis caused by NETs is more likely to cause severe emergency symptoms, including mesenteric angina and ischemia, venous congestion, and intestinal obstruction [7]. Thus, in 2017, the North American Society for Neuroendocrine Oncology recommended primary tumor resection for patients with stage IV small bowel NETs to avoid local complications and improve the quality of life [8]. Moreover, the long-term efficacy of local resection, such as endoscopic treatment, for intestinal NETs was not inferior to that of radical bowel resection [9]. However, it is debatable whether excision of primary tumors and metastases improves survival rates for patients with stage IV small bowel NETs [10]. Therefore, it is necessary to explore the impact of surgical treatment on the survival of patients with metastatic small bowel cancers, including adenocarcinomas and NETs.

The Surveillance, Epidemiology, and End Results (SEER) database collects data on cancer diagnosis, treatment, and survival across states in the United States (~30% of the population); it is a national project led by the National Cancer Center and partnering with state cancer registries [11]. Although there have been studies of small bowel metastatic tumors in the SEER database, they mainly explored the patterns of metastasis and survival analysis, as well as the efficacy of surgery and chemotherapy, and did not analyze the impact of different surgical methods on the prognosis [12, 13]. Therefore, this study, based on the SEER database, conducted propensity score–matching (PSM) analysis to explore the effects of different primary tumor resections and metastatic operations on the survival rates of metastatic small bowel cancer.

## Materials and methods

### Patient population

**Fig 1:**
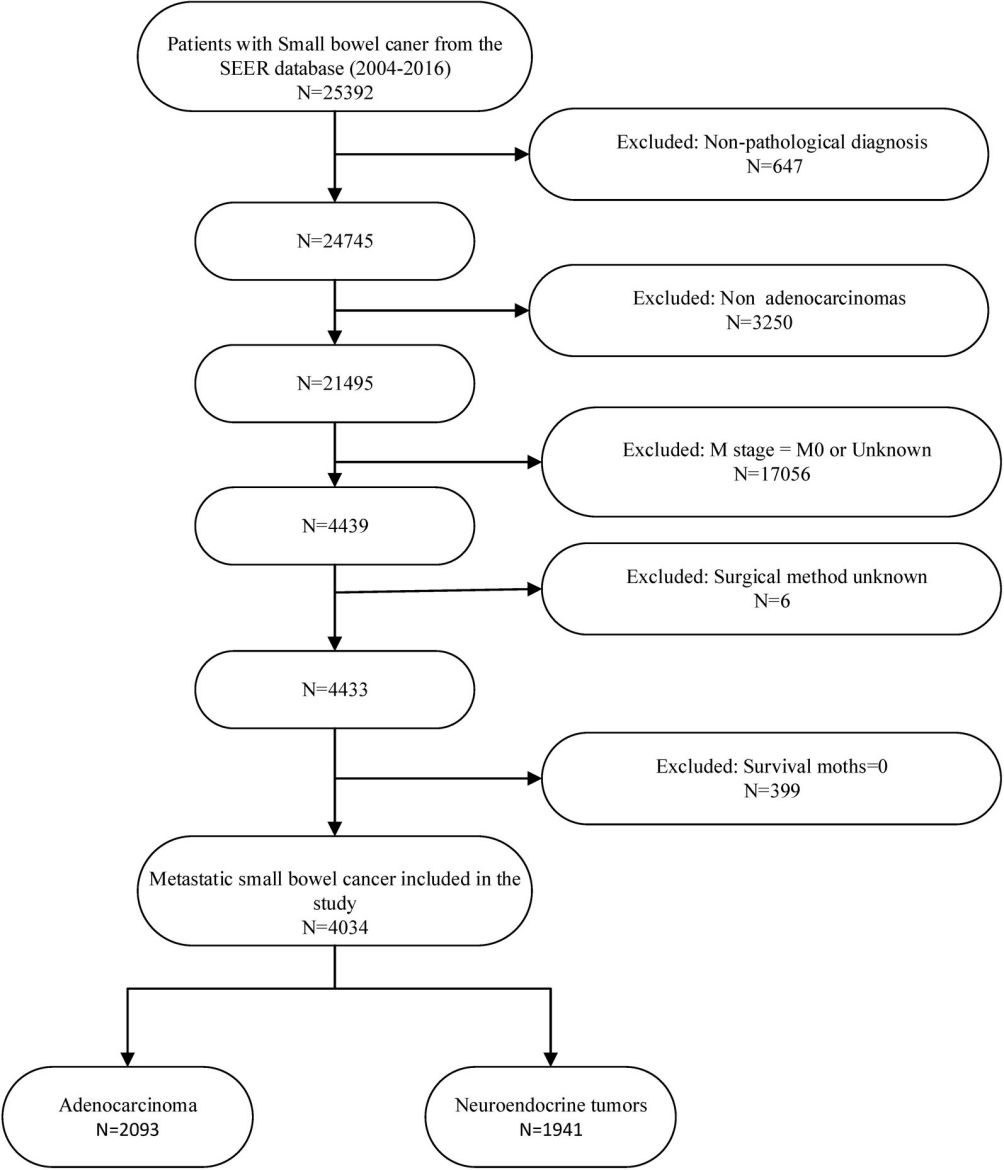
Inclusion and exclusion procedures for metastatic small bowel cancer patients from SEER database.

We explored the SEER*Stat software to obtain detailed clinical and survival data for patients from the version released in April 2019. The patients were mainly those with diagnoses of small bowel cancer from 2004 to 2016. The information we extracted included basic data (i.e., age, sex, race, marital status, and insurance recode), detailed clinicopathologic data (i.e., grade, histology, TNM), follow-up information, and therapy options. The regional nodes examined (RNE) were either fewer than eight or eight or more, according to the NCCN guidelines [2]. We classified the operation targeting the primary tumor site (i.e., the small intestine) as localized surgery or intestine-ectomy based on previous studies and experience with colorectal cancer [9, 14]. Localized surgery is the use of photodynamic therapy, electrocautery, or laser procedures to destroy or excise a local tumor or for the simple/partial removal of a tumor from the bowel lining. Intestine-ectomy is defined as the removal of the segment of intestine in which the primary tumor is located or an operation of large scope (plus the removal of contiguous organs), regardless of whether local lymph nodes are removed. Surgical methods for primary tumors were coded according to the RX Summ–Surg Prim Site (1998+) code of 10 to 30 for localized surgery and 40 to 70 for intestine-ectomy. The inclusion criteria of this study were as follows: (a) pathologic diagnosis of small bowel cancer only; (b) confirmed synchronous distance metastasis; (c) definite information on the surgical procedure (RX Summ–Surg Prim Site [1998+] code of 0 to 70); (d) survival time was definite and longer than 0 months; (e) pathologic typing of adenocarcinoma or NETs (histology recode–broad groupings code of 8140 to 8389 or 8440 to 8499); and (f) clear ultimate cause of death. The detailed patient screening process is shown in **Fig 1**.

This study was based on a retrospective analysis of information from the SEER database and used no identifiable patient information; the information was anonymous. Therefore, written informed consent was not required in this study. The study was based on the ethical standards of the Helsinki Declaration, as well as national and international norms, and was reviewed and approved by the Ethics Committee of Xiangya Hospital of Central South University in Changsha, Hunan, China.

### Statistical analysis

This study defined the overall survival (OS) rate and cancer-specific survival (CSS) rate as the main endpoints. The OS time referred to the time between the diagnosis of small bowel cancer and death for any reason. The CSS time was the time from the diagnosis of small bowel cancer to death due to that cancer. The log-rank test was used to estimate survival differences, and the Kaplan–Meier analysis was performed to compare survival rates. Univariate and multivariate Cox proportional hazards regression models were utilized to determine the independent prognostic factors in metastatic small bowel cancer patients. The 1:1 PSM was performed to analyze the effect of primary tumor resection, metastasis resection, and different surgical methods on the prognosis. SPSS 26.0 software was used for all analysis processes, and a *p*-value of less than .05 was determined to be statistically significant.

## Results

### Basic characteristics

Of 25,392 small bowel cancer patients reviewed, 4,034 patients, including 2,093 mSIA patients and 1,941 mSI-NETs patients, met the selection criteria for further analysis **(Table 1)**. Approximately 38.70% of patients with mSIA underwent resection of the primary tumor, and 17.53% underwent surgery for metastases. The proportion of patients undergoing primary tumor resection was as high as 76.09%, and metastasis resection accounted for 33.90% in patients with small intestinal NETs. Liver metastases accounted for 40.41% of the mSIAs and 62.96% of the mSI-NETs in the period from 2010 to 2016.

**Table 1:** The basic clinicopathologic features of patients with metastatic small bowel cancer.

Characteristics	Level	Adenocarcinoma (n = 2093)	Neuroendocrine tumors (n = 1941)
		Number (%)	Number (%)
Insurance recode	No/unknown	650 (31.06%)	308 (15.87%)
	Insured	1443 (68.94%)	1633 (84.13%)
Marital status	Single/unknown	927 (44.29%)	757 (39.00%%)
	Married	1166 (55.71%)	1184 (61.00%)
Race	Nonwhite	591 (28.24%)	296 (15.25%)
	White	1502 (71.76%)	1645 (84.75%)
Age	<60 years	630 (30.10%)	751 (38.69%)
	≥60 years	1463 (69.90%)	1190 (61.31%)
Sex	Female	979 (46.77%)	967 (49.82%)
	Male	1114 (53.23%)	974 (50.18%)
Primary tumor site	Duodenum	1223 (58.43%)	136 (7.01%)
	Jejunum and ileum	533 (25.47%)	1088 (56.05%)
	Unknown	337 (16.10%)	717 (36.94%)
Grade	I	88 (4.21%)	1013 (52.19%)
	II	710 (33.92%)	331 (17.05%)
	III/IV	664 (31.72%)	83 (4.28%)
	Unknown	631 (30.15%)	514 (26.48%)
T stage	T1–2	268 (12.81%)	249 (12.83%)
	T3	348 (16.63%)	747 (38.49%)
	T4	853 (40.75%)	588 (30.29%)
	Unknown	624 (29.81%)	357 (18.39%)
N stage	N0	837 (39.99%)	499 (25.71%)
	N1–2	881 (42.09%)	1262 (65.02%)
	Unknown	375 (17.92%)	180 (9.27%)
Surgery of primary tumor	No surgery	1283 (61.30%)	464 (23.91%)
	Localized surgery	467 (22.31%)	834 (42.97%)
	Intestine-ectomy	343 (16.39%)	643 (33.12%)
Surgery of metastatic disease	No/unknown	1726 (82.47%)	1283 (66.10%)
	Yes	367 (17.53%)	658 (33.90%)
Chemotherapy	No/unknown	838 (40.04%)	1611 (83.00%)
	Yes	1255 (59.96%)	330 (17.00%)
Regional nodes examined	<8	1673 (79.93%)	1003 (51.67%)
	≥8	328 (15.67%)	848 (43.69%)
	Unknown	92 (4.40%)	90 (4.64%)
Tumor size	<5 cm	714 (34.11%)	1386 (71.41%)
	≥5 cm	399 (19.06%)	143 (7.37%)
	Unknown	980 (46.83%)	412 (21.22%)
Years of diagnosis	2004–2009	811 (38.75%)	259 (13.34%)
	2010–2016	1282 (61.25%)	1682 (86.66%)
Metastatic site*	Liver	518 (40.41%)	1059 (62.96%)
	Lung	197 (15.37%)	84 (4.99%)
	Brain and bone	157 (12.25%)	117 (6.96%)
	Unknown	349 (27.22%)	422 (25.09%)

* Note: Due to the lack of relevant data on metastatic sites before 2010, the number and proportion of cases at each metastatic site were calculated from 2010.

### Survival analysis before PSM

**Fig 2:**
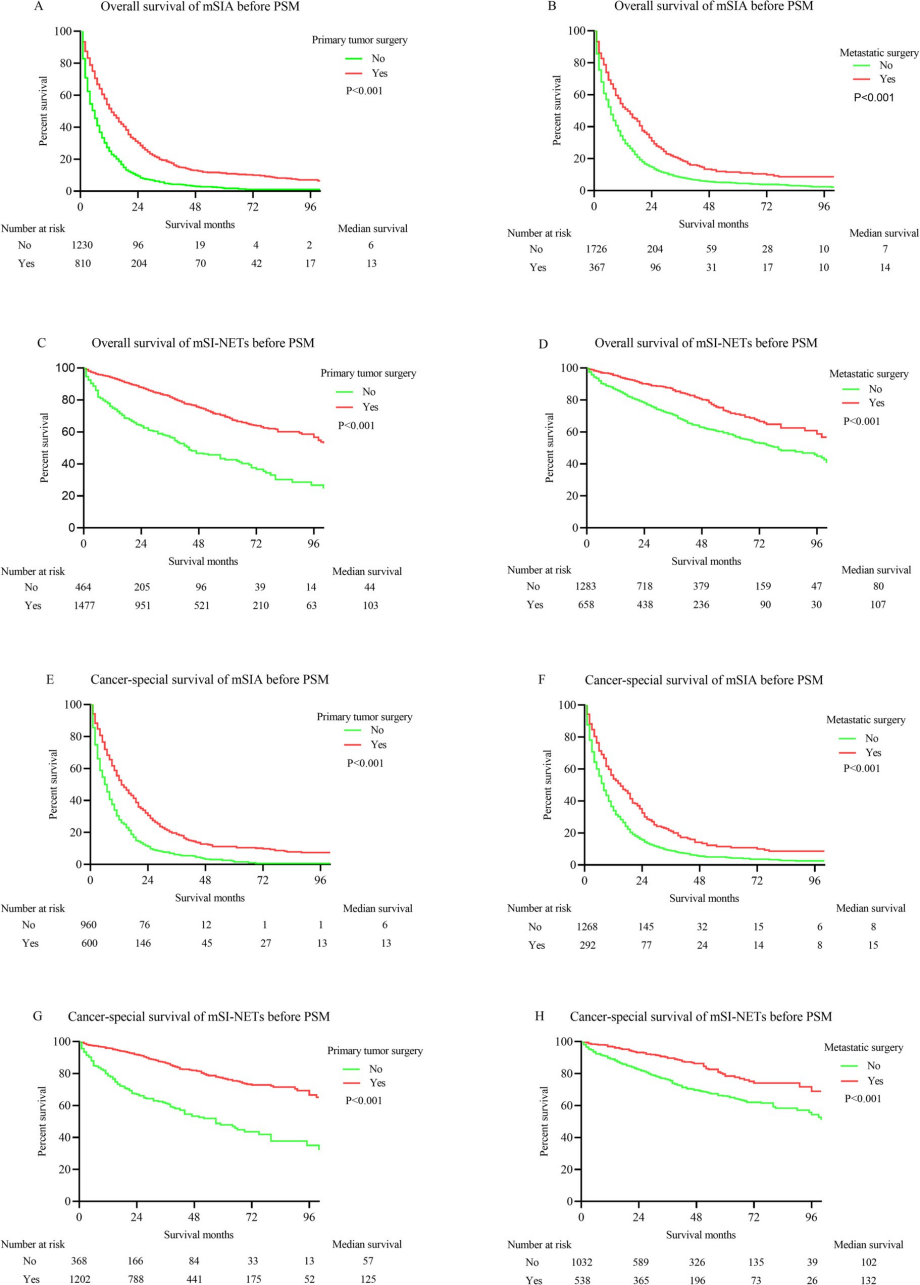
K-M curves for OS and CSS in metastatic small bowel cancer patients before PSM. (A) OS of mSIA with and without primary tumor surgery. (B) OS of mSIA with and without metastatic surgery. (C) OS of mSI-NETs with and without primary tumor surgery. (D) OS of mSI-NETs with and without metastatic surgery. (E) CSS of mSIA with and without primary tumor surgery. (F) CSS of mSIA with and without metastatic surgery. (G) CSS of mSI-NETs with and without primary tumor surgery. (H) CSS of mSI-NETs with and without metastatic surgery.

The K-M survival analysis showed a significant dissimilarity in the OS time between patients with and without primary tumor surgery, which included localized surgery and intestine-ectomy (*p* < .001; **Fig 2A**). The median OS time of patients undergoing primary tumor surgery was 13 months, compared with a 6-month median OS time for patients without surgery. Similarly, metastatic surgery was able to improve the OS for mSIAs (*p* < .001; **Fig 2B**). The median OS times in mSIA patients with and without metastatic resection were 14 months and 7 months, respectively. The K-M survival analysis showed that the mSI-NETs patients who underwent primary tumor resection and metastatic operation had a significantly superior OS time to those who did not undergo those procedures (primary tumor surgery: *p* < .001; **Fig 2C;** metastatic operation: *p* < .001; **Fig 2D**). The median OS times were 103 months for mSI-NETs patients with primary tumor surgery, 44 months for patients without that procedure, 107 months for patients with metastatic surgery, and 80 months for mSI-NETs patients who missed metastasis resection.

The K-M survival analysis then was adopted to display the effect of primary tumor surgery and metastatic operation on the CSS times of patients with mSIA and mSI-NETs. Both primary tumor surgery and metastatic operation improved the CSS times for mSIA and mSI-NETs patients (primary tumor surgery for mSIA: *p* < .001, **Fig 2E;** metastatic surgery for mSIA: *p* < .001, **Fig 2F;** primary tumor surgery for mSI-NETs: *p* < .001, **Fig 2G;** metastatic surgery for mSI-NETs: *p* < .001, **Fig 2H**). Meanwhile, primary tumor surgery and metastatic surgery extended the 7-month median CSS time for mSIA patients (primary tumor surgery: 13 months vs. 6 months; metastatic surgery: 15 months vs. 8 months). A primary tumor operation was able to prolong the 68-month median CSS time (125 months vs. 57 months), and metastatic surgery extended the 30-month median CSS time for mSI-NETs (132 months vs. 102 months).

We further explored the independent prognostic factors for OS and CSS in patients with mSIA and mSI-NETs. Marital status, age, and metastatic surgery were significantly related to the OS, but they could not serve as independent prognostic factors for the CSS for mSIA (**Table 2**). The only independent prognostic factor that differed between the OS and CSS was the marital status for mSI-NETs (**Table 3**). Importantly, primary tumor surgery and metastatic operation could serve as independent prognostic factors, except that metastatic operation could not be used as an independent prognostic factor for the CSS in mSIA (*p* = .074). Moreover, chemotherapy was a poor prognostic factor for mSI-NETs (*p* < .001 for both OS and CSS).

**Table 2:** Univariate and multivariate analysis for OS and CSS of patients with mSIA.

Characteristics	Level	OS				CSS			
		Univariate analysis	Multivariate analysis			Univariate analysis	Multivariate analysis		
		*P*	HR	95%CI	*P*	*P*	HR	95%CI	*P*
Insurance		0.809				0.334			
	No/unknown								
	Insured								
Marital status		0.001			0.041	0.040			0.207
Single/unknown			Reference	Reference	Reference		Reference	Reference	Reference
Married			0.907	0.825–0.996	0.041		0.931	0.832–1.041	0.207
Race		0.329				0.873			
	Nonwhite								
	White								
Age		<0.001			<0.001	0.001			0.820
	<60 years		Reference	Reference	Reference		Reference	Reference	Reference
	≥60 years		1.290	1.159–1.436	<0.001		1.014	0.898–1.145	0.820
Sex		0.868				0.903			
	Female								
	Male								
Grade		<0.001			<0.001	<0.001			<0.001
	I		Reference	Reference	Reference		Reference	Reference	Reference
	II		1.091	0.848–1.403	0.498		1.021	0.761–1.370	0.889
	III/IV		1.511	1.174–1.944	0.001		1.501	1.119–2.014	0.007
	Unknown		1.080	0.836–1.396	0.555		1.040	0.771–1.403	0.797
Primary tumor site		<0.001			0.018	<0.001			0.009
Duodenum			Reference	Reference	Reference		Reference	Reference	Reference
Jejunum and ileum			0.820	0.715–0.941	0.005		0.778	0.661–0.916	0.003
Unknown			0.900	0.778–1.042	0.159		0.851	0.714–1.013	0.070
T stage		<0.001			0.002	<0.001			0.001
	T1-2		Reference	Reference	Reference		Reference	Reference	Reference
	T3		0.853	0.708–1.028	0.095		0.871	0.697–1.090	0.228
	T4		1.111	0.952–1.296	0.180		1.203	1.000–1.447	0.050
	Unknown		1.109	0.950–1.295	0.190		1.165	0.966–1.404	0.111
N stage		0.051				0.069			
	N0								
	N1-2								
	Unknown								
Surgery of primary tumor		<0.001			<0.001	<0.001			<0.001
	No surgery		Reference	Reference	Reference		Reference	Reference	Reference
Localized surgery			0.743	0.627–0.880	0.001		0.709	0.579–0.868	0.001
Intestine-ectomy			0.627	0.521–0.754	<0.001		0.616	0.495–0.765	<0.001
Surgery of metastatic disease		<0.001			0.004	<0.001			0.074
	No/unknown		Reference	Reference	Reference		Reference	Reference	Reference
	Yes		0.822	0.720–0.938	0.004		0.870	0.746–1.014	0.074
Chemotherapy		<0.001			<0.001	<0.001			<0.001
	No/unknown		Reference	Reference	Reference		Reference	Reference	Reference
	Yes		0.556	0.505–0.614	<0.001		0.623	0.554–0.702	<0.001
Regional nodes examined		<0.001			0.227	<0.001			0.306
	<8		Reference	Reference	Reference		Reference	Reference	Reference
	≥8		0.867	0.736–1.022	0.089		0.876	0.721–1.064	0.183
	Unknown		1.010	0.799–1.275	0.936		0.872	0.651–1.167	0.357
Tumor size		<0.001			<0.001	<0.001			<0.001
	<5 cm		Reference	Reference	Reference		Reference	Reference	Reference
	≥5 cm		1.286	1.122–1.473	<0.001		1.386	1.182–1.626	<0.001
	Unknown		1.258	1.110–1.426	<0.001		1.264	1.088–1.468	0.002
Metastatic site		<0.001			<0.001	<0.001			<0.001
	Liver		Reference	Reference	Reference		Reference	Reference	Reference
	Lung		0.712	0.581–0.873	0.001		0.700	0.547–0.895	0.005
	Brain or bone		1.026	0.843–1.249	0.796		1.119	0.890–1.407	0.336
	Unknown		0.793	0.707–0.891	<0.001		0.796	0.694–0.914	0.001

**Table 3:** Univariate and multivariate analysis for OS and CSS of patients with mSI-NETs.

Characteristics	Level	OS				CSS			
		Univariate analysis	Multivariate analysis			Univariate analysis	Multivariate analysis		
		*P*	HR	95%CI	*P*	*P*	HR	95%CI	*P*
Insurance		0.019			0.209	0.048			0.274
	No/unknown		Reference	Reference	Reference		Reference	Reference	Reference
	Insured		0.874	0.709–1.078	0.209		0.863	0.662–1.124	0.274
Marital status		<0.001			0.006	0.028			0.104
Single/unknown			Reference	Reference	Reference		Reference	Reference	Reference
Married			0.786	0.663–0.932	0.006		0.835	0.672–1.038	0.104
Race		0.689				0.230			
	Nonwhite								
	White								
Age		<0.001			<0.001	<0.001			<0.001
	<60 years		Reference	Reference	Reference		Reference	Reference	Reference
	≥60 years		2.234	1.837–2.716	<0.001		1.731	1.368–2.190	<0.001
Sex		0.055				0.579			
	Female								
	Male								
Grade		<0.001			<0.001	<0.001			<0.001
	I		Reference	Reference	Reference		Reference	Reference	Reference
	II		1.541	1.193–1.991	0.001		1.520	1.085–2.130	0.015
	III/IV		4.240	3.141–5.722	<0.001		4.950	3.428–7.149	<0.001
	Unknown		1.502	1.220–1.848	<0.001		1.557	1.195–2.028	0.001
Primary tumor site		0.672				0.791			
Duodenum									
Jejunum and ileum									
Unknown									
T stage		<0.001			0.044	<0.001			0.026
	T1-2		Reference	Reference	Reference		Reference	Reference	Reference
	T3		1.347	0.985–1.842	0.063		1.603	1.043–2.462	0.031
	T4		1.474	1.069–2.031	0.018		1.823	1.179–2.818	0.007
	Unknown		1.042	0.715–1.517	0.831		1.210	0.740–1.980	0.447
N stage		0.033			0.283	<0.001			0.368
	N0		Reference	Reference	Reference		Reference	Reference	Reference
	N1-2		0.984	0.793–1.222	0.884		1.008	0.763–1.331	0.958
	Unknown		1.238	0.927–1.654	0.149		1.276	0.894–1.823	0.179
Surgery of primary tumor		<0.001			<0.001	<0.001			<0.001
	No surgery		Reference	Reference	Reference		Reference	Reference	Reference
Localized surgery			0.509	0.378–0.685	<0.001		0.400	0.273–0.584	<0.001
Intestine-ectomy			0.566	0.412–0.777	<0.001		0.500	0.335–0.746	0.001
Surgery of metastatic diseases		<0.001			0.020	<0.001			0.032
	No/unknown		Reference	Reference	Reference		Reference	Reference	Reference
	Yes		0.785	0.640–0.963	0.020		0.746	0.570–0.976	0.032
Chemotherapy		<0.001			<0.001	<0.001			<0.001
	No/unknown		Reference	Reference	Reference		Reference	Reference	Reference
	Yes		1.643	1.350–2.000	<0.001		1.746	1.367–2.230	<0.001
RNE		<0.001			0.089	<0.001			0.317
	<8		Reference	Reference	Reference		Reference	Reference	Reference
	≥8		0.790	0.629–0.992	0.043		0.822	0.611–1.106	0.195
	Unknown		0.801	0.554–1.158	0.238		0.789	0.492–1.267	0.327
Tumor size		<0.001			0.002	<0.001			<0.001
	<5 cm		Reference	Reference	Reference		Reference	Reference	Reference
	≥5 cm		1.601	1.210–2.118	0.001		1.980	1.416–2.767	<0.001
	Unknown		1.333	1.023–1.737	0.033		1.365	0.976–1.909	0.069
Metastatic site		0.073				0.635			
	Liver								
	Lung								
	Brain or bone								
	Unknown								

### Survival analysis after PSM

**Fig 3:**
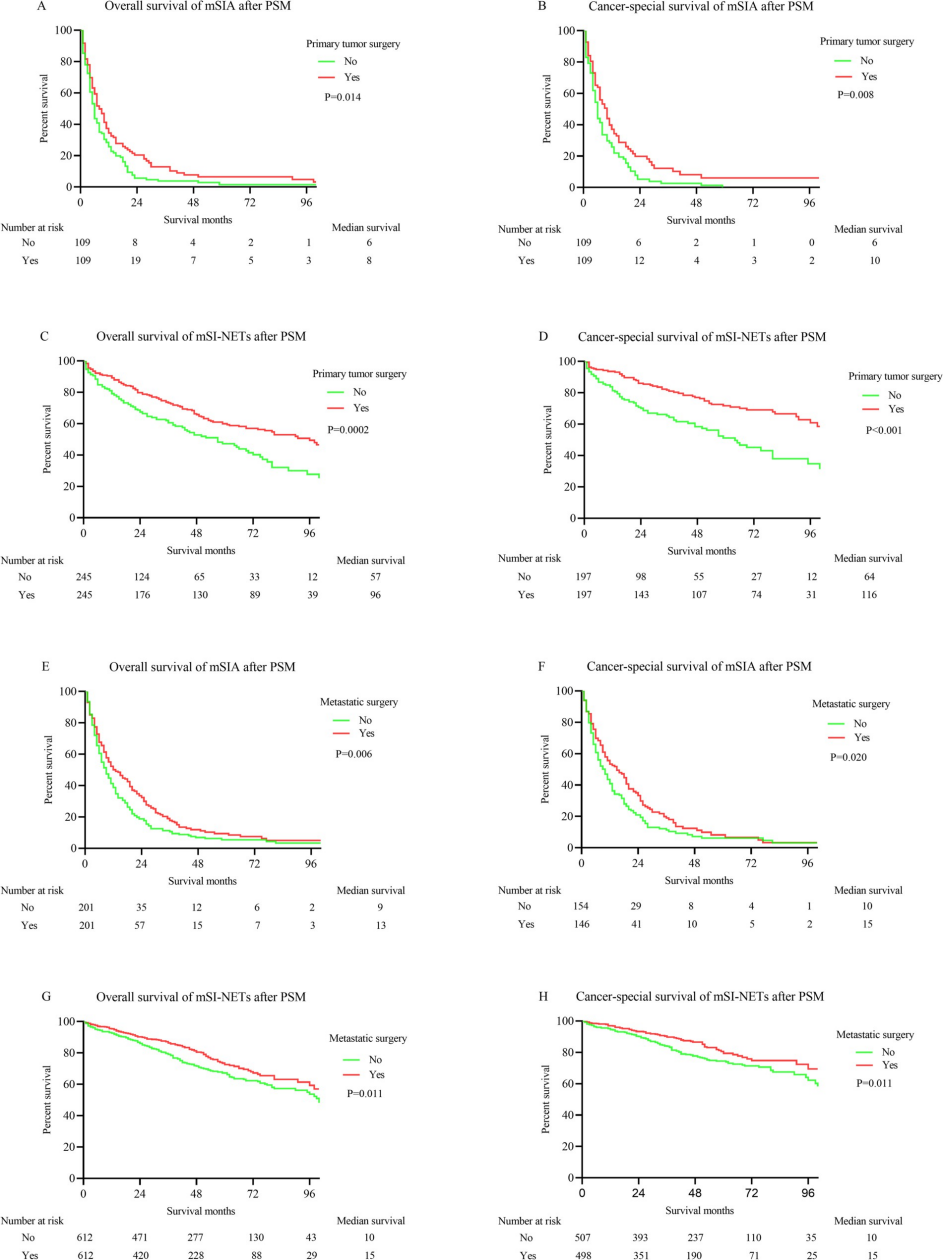
K-M curves for OS and CSS in metastatic small bowel cancer patients after PSM. (A) OS of mSIA with and without primary tumor surgery. (B) CSS of mSIA with and without primary tumor surgery. (C) OS of mSI-NETs with and without primary tumor surgery. (D) CSS of mSI-NETs with and without primary tumor surgery. (E) OS of mSIA with and without metastatic surgery. (F) CSS of mSIA with and without metastatic surgery. (G) OS of mSI-NETs with and without metastatic surgery. (H) CSS of mSI-NETs with and without metastatic surgery.

PSM was performed to eliminate the impact of other variables on the survival time. The characteristics of patients before and after PSM are summarized in **S1–S8 Tables**. PSM effectively abolished the difference between the two groups. The K-M survival analysis was utilized to compare the impact of surgery on survival time, and it was cause for optimism about the effects of surgery: **Fig 3A** and **3B**: effects of primary tumor surgery for mSIA on OS (*p* = .014) and CSS (*p* = .008); **Fig 3C** and **3D**: effects of primary tumor surgery for mSI-NETs on OS (*p* = .0002) and CSS (*p* < .001); **Fig 3E** and **3F**: effects of metastatic operation for mSIA on OS (*p* = .006) and CSS (*p* = .020); **Fig 3G** and **3H**: effects of metastatic operation for mSI-NETs on OS (*p* = .011) and CSS (*p* = .011). Interestingly, metastatic operation, a non–independent prognostic factor for CSS in the Cox regression model, was confirmed to have a positive effect on survival in patients with mSIA.

### Localized surgery or intestine-ectomy

**Fig 4:**
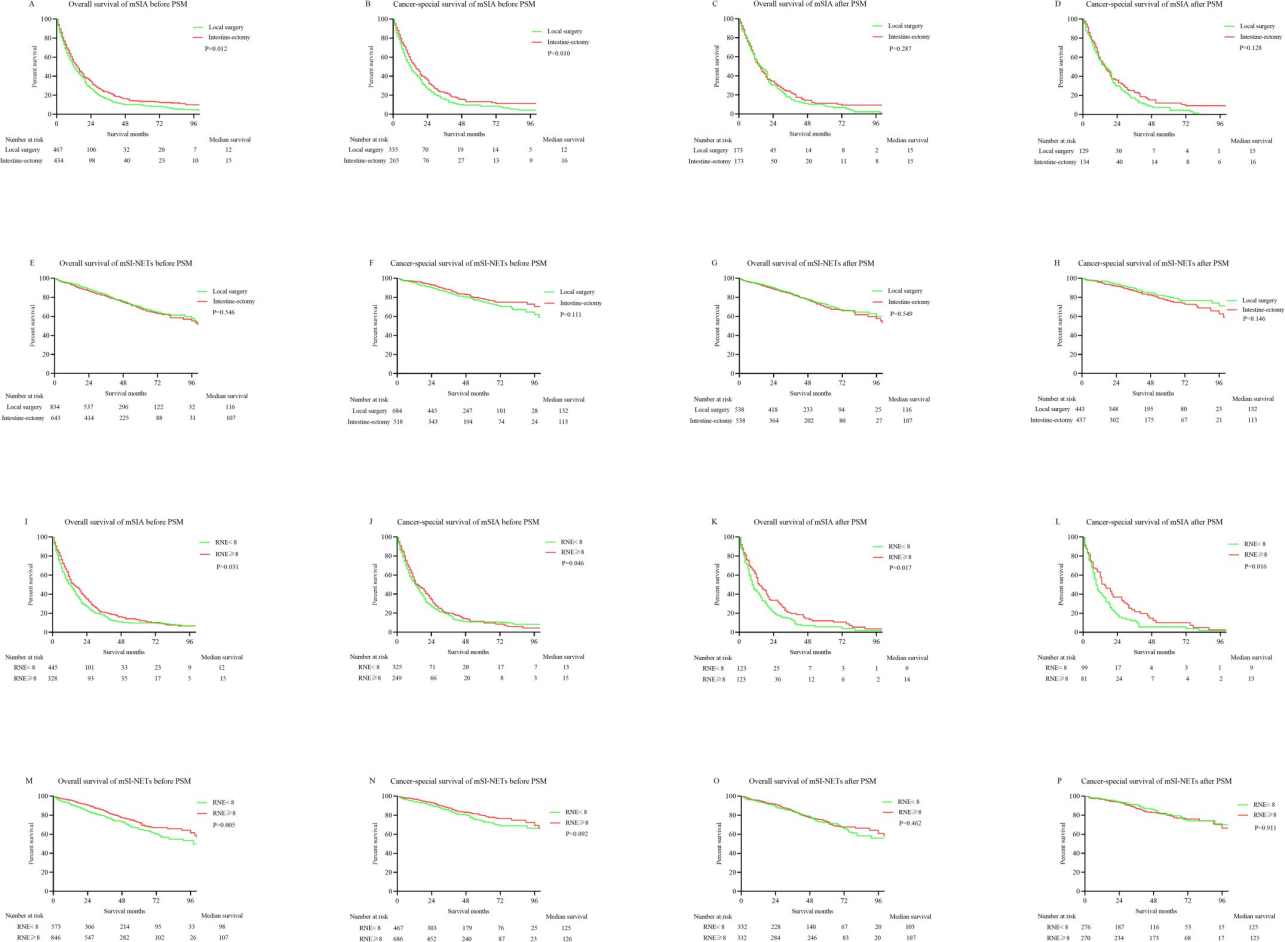
K-M curves for OS and CSS in metastatic small bowel cancer patients undergoing different types of primary tumor surgery (local surgery vs. intestine-ectomy) and RNE (RNE <8 vs RNE ≥8) before and after PSM. (A) OS of mSIA before PSM. (B) CSS of mSIA before PSM. (C) OS of mSIA after PSM. (D) CSS of mSIA after PSM. (E) OS of mSI-NETs before PSM. (F) CSS of mSI-NETs before PSM. (G) OS of mSI-NETs after PSM. (H) CSS of mSI-NETs after PSM. (I) OS of mSIA before PSM. (J) CSS of mSIA before PSM. (K) OS of mSIA after PSM. (L) CSS of mSIA after PSM. (M) OS of mSI-NETs before PSM. (N) CSS of mSI-NETs before PSM. (O) OS of mSI-NETs after PSM. (P) CSS of mSI-NETs after PSM.

Primary tumor surgery improved the survival time for mSIA and mSI-NETs. We planned to further explore the effect of surgical details on survival for mSIA and mSI-NETs. Intestine-ectomy seemed to be better than localized surgery in improving both the OS time (*p* = .012) and CSS time (*p* = .010) for mSIA. However, the survival difference disappeared after PSM was used (OS: *p* = .287; CSS: *p* = .128). Intestine-ectomy did not provide a survival benefit in either the OS or CSS time for mSI-NETs compared with localized surgery before and after the PSM (before: OS *p* = .546; CSS *p* = .111; after: OS *p* = .549; CSS *p* = .146; **Fig 4A–4H**).

RNE was considered the priority for the assessment of the quality of surgery [15, 16]. Therefore, its effect on the survival times was also investigated for mSIA and mSI-NETs. An increased RNE was able to improve the OS (*p* = .031) and CSS (*p* = .046) before PSM for mSIA. The survival differences became more significant after PSM (OS: *p* = .017; CSS: *p* = .016). However, the survival difference was seen only in the OS analysis before PSM for mSI-NETs (*p* = .005). Therefore, regional lymph node dissection was not necessary for mSI-NETs (**Fig 4I–4P**).

### Therapeutic strategy for mSIA and mSI-NETs

**Fig 5:**
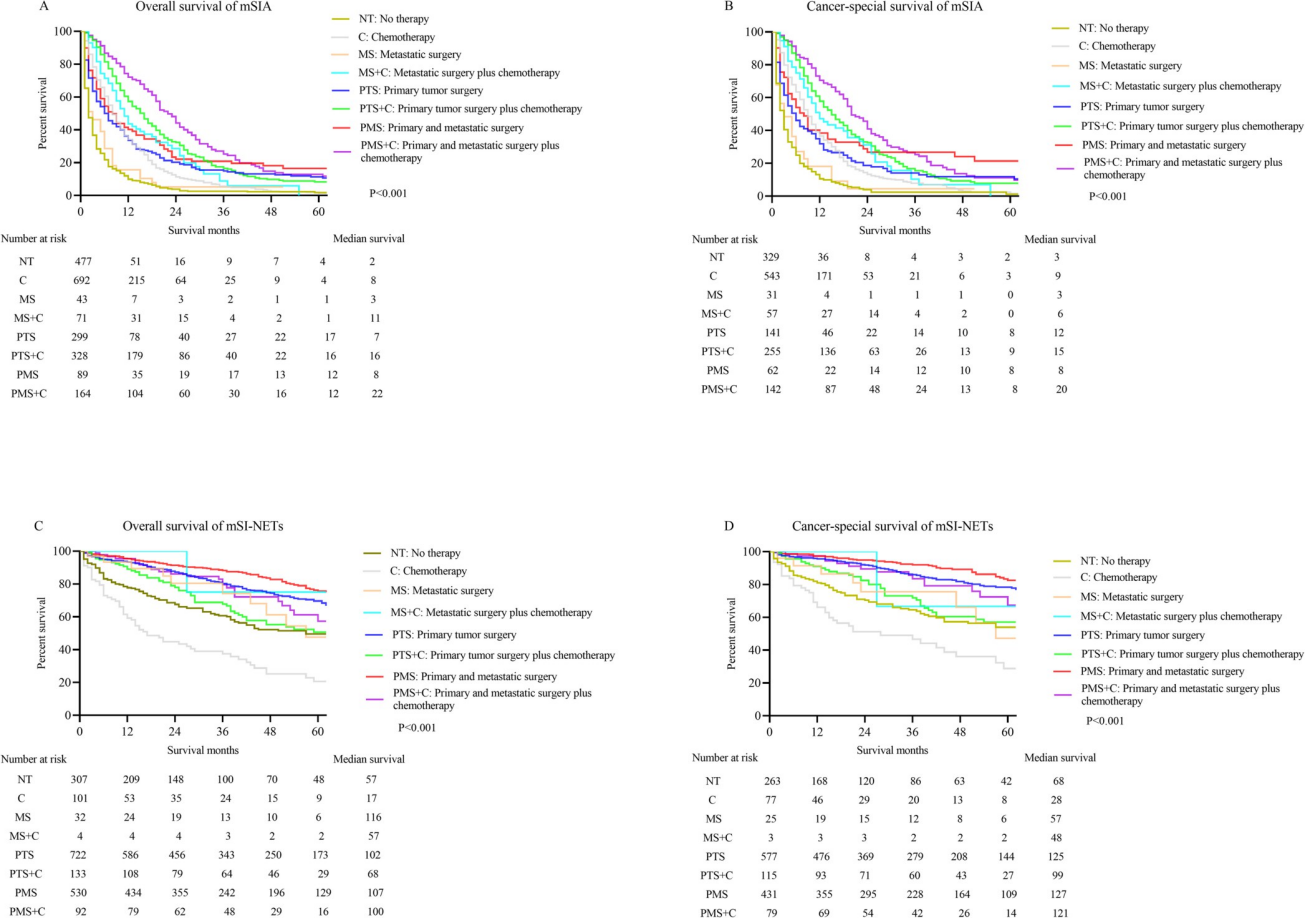
K-M curves for OS and CSS in metastatic small bowel cancer patients who received different treatment regimens. (A) OS of mSIA. (B) CSS of mSIA. (C) OS of mSI-NETs. (D) CSS of mSI-NETs.

The patients were stratified according to therapeutic strategy to explore the optimal treatment options for mSIA and mSI-NETs (**Fig 5**). The best therapeutic strategy was primary and metastatic excision plus chemotherapy for mSIA (median OS: 22 months; median CSS: 20 months) and primary and metastatic excision for mSI-NETs (median OS: 117 months; median CSS: 126 months). Furthermore, chemotherapy was essential for mSIA because it provided significantly improved survival times (median OS: chemotherapy vs. no therapy: 8 months vs. 2 months; metastatic surgery plus chemotherapy vs. metastatic surgery: 11 months vs. 3 months; primary tumor surgery plus chemotherapy vs. primary tumor surgery: 16 months vs. 7 months; primary and metastatic excision plus chemotherapy vs. primary and metastatic excision: 22 months vs. 8 months; median CSS: chemotherapy vs. no therapy: 9 months vs. 3 months; metastatic surgery plus chemotherapy vs. metastatic surgery: 12 months vs. 3 months; primary tumor surgery plus chemotherapy vs. primary tumor surgery: 15 months vs. 6 months; primary and metastatic excision plus chemotherapy vs. primary and metastatic excision: 20 months vs. 8 months). Contrarily, chemotherapy decreased the survival time for mSI-NETs (median OS: chemotherapy vs. no therapy: 17 months vs. 57 months; metastatic surgery plus chemotherapy vs. metastatic surgery: 20 months vs. 57 months; primary tumor surgery plus chemotherapy vs. primary tumor surgery: 68 months vs. 116 months; primary and metastatic excision plus chemotherapy vs. primary and metastatic excision: 90 months vs. 117 months; median CSS: chemotherapy vs. no therapy: 28 months vs. 66 months; metastatic surgery plus chemotherapy vs. metastatic surgery: 30 months vs. 57 months; primary tumor surgery plus chemotherapy vs. primary tumor surgery: 99 months vs. 125 months; primary and metastatic excision plus chemotherapy vs. primary and metastatic excision: 100 months vs. 126 months).

## Discussion

In this study, we identified the positive effect of surgery, including primary tumor resection and metastatic operation, on the survival of patients with metastatic small bowel cancers. A considerable part of the primary tumor is diagnosed after the metastatic appearance in mSIA and mSI-NETs patients due to the insidious progress and the lack of specific tumor markers [17]. Small intestinal adenocarcinomas and NETs, as the two most common pathologic types of small intestinal cancers, should be analyzed together because for them, there may be a lack of pathologic tissue available that could affirm the pathologic type before surgery. This study also explored the differences in the effects of surgery on the survival times of mSIA and mSI-NETs.

Surgery is undoubtedly the most preferred option for curing various locoregional gastrointestinal tumors, and it significantly improves the survival times of patients with such tumors [18]. However, there is currently no consensus about which surgical treatment is best for metastatic small bowel cancers. The latest version of the guidelines, which recommends surgical resection only for those with tumor-related complications [2], is not agreed upon by all surgeons. We insist that patients with metastatic small bowel cancer should be evaluated for the possibility of surgical resection at any time, rather than resected as a last resort. In order to promote this recommendation of surgical treatment for metastatic small bowel cancer, we used information from the SEER database; we explored the effects of primary or metastatic resection on survival outcomes for patients with metastatic small bowel cancer. In doing so, it was necessary to determine the differences between the treatment of mSIA and mSI-NETs. Firstly, chemotherapy improved the survival times of mSIA patients but did not provide survival benefits for mSI-NETs patients. Previous studies have shown that cytotoxic chemotherapy plays a limited role in the treatment of well-differentiated SI-NETs, and even has significant toxicity in patients [19, 20]. However, the number of neuroendocrine cancers (4.28%) in this study was very small, so it is impossible to further discuss the effect of chemotherapy on neuroendocrine cancer based on tumor grade. We could only infer that chemotherapy did not seem to provide survival benefits for well-differentiated NET patients, excluding neuroendocrine cancer. In addition, adequate regional lymph node dissection was necessary for mSIA but not for mSI-NETs, and localized surgery was enough for appropriate mSI-NETs patients (who did not have neuroendocrine cancer). It is worth noting that inadequate lymph node dissection could not improve the prognosis of mSIA compared with localized surgery. Hence, localized surgery for selected mSIA patients may be an alternative option to intestinal resection with inadequate lymph node dissection.

Several retrospective studies and literature reviews reported that removal of the primary tumor could prolong the survival of stage IV small bowel cancer patients [21–23]. Moreover, experiences from the treatment of colorectal cancer demonstrated that survival benefits could be achieved by performing primary tumor surgery in the presence of metastatic disease [24]. However, the mechanism by which primary tumor surgery prolongs survival in patients with distant metastases remains unclear. The repair of the immune system after primary tumor surgery may be one of the causes [25, 26]. Patients with metastatic gastrointestinal tumors often have an increased neutrophil and lymphocyte ratio, which is regarded as a sign of systemic inflammation [27–29]. Systemic inflammation contributes to tumor survival and development through the presence of abnormal T cells and the loss of immune cytotoxic function [30, 31]. The excision of primary tumors may reduce systemic inflammation, revive the immune function, and improve the prognosis for patients with metastatic disease [32]. Adequate lymph node dissection may also be associated with tumor immunity and thereby improve the prognosis for metastatic disease. Foxp3+ regulatory T cells in draining lymph nodes contribute to tumor development and may lead to CD8+ T-cell incompetence in colorectal cancer [33]. Dissection of enough lymph nodes can reduce the impact of immunosuppression on the tumor microenvironment, thus improving survival. In cases where the removal of enough lymph nodes does not provide survival benefits for mSI-NETs, it may be because lymph node metastasis is one of the major metastatic patterns in patients with adenocarcinoma but not in patients with NETs [34]. This study did not further discuss the effect of lymph node dissection on survival for endocrine cancers, which have a higher rate of lymph node metastasis than NETs [35], owing to the scarcity of cases. It has been suggested that the persistence of primary tumors may increase the level of circulating tumor cells (CTCs), which can cause micrometastases and which eventually inevitably develop into metastases in the liver, lung, or other sites [36, 37]. A previous study based on the current epidemiologic analysis reported that almost all distant metastases occurred before the primary tumor was removed and that the metastases themselves produced no other metastases [38]. Therefore, the survival benefit after removal of the primary tumor may be due to a reduction in the number of CTCs [26].

Like other gastrointestinal tumors, small intestinal tumors commonly metastasize to the liver [39]. The proportion of liver metastases was 40.41% in mSIA patients and 62.96% in mSI-NETs patients diagnosed from 2010 to 2016 in this study (metastatic information was not provided by the SEER database for the period of 2004 to 2009). A previous study showed that the resection of metastases could improve the survival of patients with metastatic NETs, especially mSI-NETs patients, leading to a 49% 5-year survival rate and 58-month median survival rate [40]. The ARCAD-NADEGE cohort study reported that the median OS of mSIA patients who underwent metastatic surgery reached 28.2 months and was significantly better than the 12.7-month median OS time for those who did not have surgery [41]. The reason for the survival benefit for patients who underwent metastasis resection may have been the reduction of the tumor burden [42].

There are some limitations in this study: (1) A retrospective analysis needs to be verified by a randomized controlled trial in the future; (2) some important information, such as data on abdominal metastases and chemotherapy regimens and the sequence of primary tumor resection and metastasis surgery, was missing in the SEER database; (3) the database did not record whether the removal of the primary tumor was performed on an emergency basis due to complications; (4) the data on neuroendocrine cancers were very minimal and could not be discussed further based on tumor grade; and (5) there was a lack of relevant tumor gene and targeted therapy information.

## Conclusions

In this study, primary tumor resection and/or metastatic lesion resection significantly improved the survival times for carefully selected patients with mSIA and mSI-NETs. Patients with mSIA needed to undergo dissection of a sufficient number of lymph nodes during surgery for primary tumors, but mSI-NETs patients did not need this step. Intestine-ectomy with adequate lymph node dissection was the optimal operation for mSIA patients with a resectable primary tumor. Localized surgery such as endoscopic resection could be a favorable choice for selected patients with mSI-NETs.

## Supporting information

S1 Table Features of patients with mSIA grouped by primary tumor surgery before and after PSM. (DOCX)

S2 Table Features of patients with mSI-NETs grouped by primary tumor surgery before and after PSM. (DOCX)

S3 Table Features of patients with mSIA grouped by metastasis surgery before and after PSM. (DOCX)

S4 Table Features of patients with mSI-NETs grouped by metastasis surgery before and after PSM. (DOCX)

S5 Table Features of patients with mSIA grouped by primary surgical approach before and after PSM. (DOCX)

S6 Table Features of patients with mSI-NETs grouped by primary surgical approach before and after PSM. (DOCX)

S7 Table Features of patients with mSIA grouped by RNE before and after PSM. (DOCX)

S8 Table Features of patients with mSI-NETs grouped by RNE before and after PSM. (DOCX)

## Abbreviations

CI: Confidence interval
CSS: Cancer-specific survival
CTCs: Circulating tumor cells
HR: Hazard ratio
mSIA: Metastatic small intestinal adenocarcinoma
mSI-NETs: Metastatic small intestinal NETs
NCCN: National Comprehensive Cancer Network
NETs: Neuroendocrine tumors
OS: Overall survival
PSM: Propensity score matching
RNE: Regional nodes examined
SEER: Surveillance, Epidemiology, and End Results
