# Supplementary material for: Survival effects of primary and metastatic surgical treatment in metastatic small intestinal tumors: A propensity score–matching study
Source: PLoS One. 2022 Jun 24;17(6):e0270608. doi: 10.1371/journal.pone.0270608 (PMC9231803; doi:10.1371/journal.pone.0270608)
Supplement: S1 Table — (DOCX) [file pone.0270608.s001.docx]

Supplementary table 1 Features of patients with mSIA grouped by primary tumor surgery before and after PSM

| Characteristics | Before PSM | | |  | After PSM | | |
| --- | --- | --- | --- | --- | --- | --- | --- |
|  | Non-surgery of primary tumor | Surgery of primary tumor | p |  | Non-surgery of primary tumor | Surgery of primary tumor | p |
| Insurance Recode |  |  | 0.029 |  |  |  | 0.271 |
| No/Unknown | 376(29.31%) | 274(33.83%) |  |  | 49(44.95%) | 41(37.61%) |  |
| Insured | 907(70.69%) | 536(66.17%) |  |  | 60(55.05%) | 68(62.39%) |  |
| Marital status |  |  | 0.288 |  |  |  | 0.567 |
| Single/Unknown | 580(45.21%) | 347(42.84%%) |  |  | 35(32.11%) | 39(35.78%) |  |
| Married | 703(54.79%) | 463(57.16%) |  |  | 74(67.89%) | 70(64.22%) |  |
| Race |  |  | 0.285 |  |  |  | 0.334 |
| Non-whites | 373(29.07%) | 218(26.91%) |  |  | 28(25.69%) | 22(20.18%) |  |
| White | 910(70.93%) | 592(73.09%) |  |  | 81(74.31%) | 87(79.82%) |  |
| Age |  |  | <0.001 |  |  |  | 0.878 |
| <60 | 301(23.46%) | 329(40.62%) |  |  | 28(25.69%) | 29(26.61%) |  |
| ≥60 | 982(76.54%) | 481(59.38%) |  |  | 81(74.31%) | 80(73.39%) |  |
| Sex |  |  | 0.727 |  |  |  | 0.055 |
| Female | 604(46.77%) | 375(49.82%) |  |  | 39(35.78%) | 53(48.62%) |  |
| Male | 679(53.23%) | 435(50.18%) |  |  | 70(64.22%) | 56(51.38%) |  |
| Primary tumor site |  |  | <0.001 |  |  |  | 0.684 |
| Duodenum | 1052(82.00%) | 171(21.11%) |  |  | 88(80.73%) | 84(77.06%) |  |
| Jejunum and Ileum | 107(8.34%) | 426(52.59%) |  |  | 10(9.17%) | 14(12.84%) |  |
| Unknown | 124(9.66%) | 213(26.30%) |  |  | 11(10.10%) | 11(10.10%) |  |
| Grade |  |  | <0.001 |  |  |  | 0.784 |
| I | 53(4.13%) | 35(4.32) |  |  | 0 | 0 |  |
| II | 361(28.14%) | 349(43.09%) |  |  | 40(36.70%) | 40(36.70%) |  |
| III/IV | 317(24.71%) | 347(42.84%) |  |  | 40(36.70%) | 44(40.37%) |  |
| Unknown | 552(43.02%) | 49(6.05%) |  |  | 29(26.60%) | 25(22.93%) |  |
| T stage |  |  | <0.001 |  |  |  | 0.997 |
| T1-2 | 232(18.08%) | 36(4.44%) |  |  | 7(6.42%) | 7(6.43%) |  |
| T3 | 95(7.40%) | 253(31.23%) |  |  | 10(9.17%) | 11(10.09%) |  |
| T4 | 375(29.24%) | 478(59.02%) |  |  | 70(64.23%) | 69(63.30%) |  |
| Unknown | 581(45.28%) | 43(5.31%) |  |  | 22(20.18%) | 22(20.18%) |  |
| N stage |  |  | <0.001 |  |  |  | 0.907 |
| N0 | 564(43.96%) | 273(33.70%) |  |  | 47(43.12%) | 45(41.28%) |  |
| N1-2 | 388(30.24%) | 493(60.87%) |  |  | 32(29.36%) | 35(32.11%) |  |
| Unknown | 331(25.80%) | 44(5.43%) |  |  | 30(27.52%) | 29(26.61%) |  |
| Metastatic operation |  |  | <0.001 |  |  |  | 0.378 |
| No/unknown | 1169(91.11%) | 557(68.77%) |  |  | 95(87.16%) | 99(90.83%) |  |
| Yes | 114(8.89%) | 253(31.23%) |  |  | 14(12.84%) | 10(9.17%) |  |
| Chemotherapy |  |  | 0.563 |  |  |  | 0.781 |
| No/Unknown | 520(40.53%) | 318(39.26%) |  |  | 43(39.45%) | 41(37.61%) |  |
| Yes | 763(59.47%) | 492(60.74%) |  |  | 66(60.55%) | 68(62.39%) |  |
| Tumor size |  |  | <0.001 |  |  |  | 0.981 |
| <5cm | 255(19.88%) | 459(56.67%) |  |  | 40(36.70%) | 39(35.78%) |  |
| ≥5cm | 189(14.73%) | 210(25.93%) |  |  | 19(17.43%) | 20(18.35%) |  |
| Unknown | 839(65.39%) | 141(17.40%) |  |  | 50(45.87%) | 50(45.87%) |  |
| Metastatic site |  |  | <0.001 |  |  |  | 0.731 |
| Liver | 395(30.79%) | 123(15.19%) |  |  | 20(18.35%) | 22(20.18%) |  |
| Lung | 130(10.13%) | 67(8.27%) |  |  | 1(0.92%) | 3(2.75%) |  |
| Brain and bone | 116(9.04%) | 41(5.06%) |  |  | 4(3.67%) | 3(2.75%) |  |
| Unknown | 642(50.04%) | 579(71.48%) |  |  | 84(77.06%) | 81(74.32%) |  |
