# Supplementary material for: Survival effects of primary and metastatic surgical treatment in metastatic small intestinal tumors: A propensity score–matching study
Source: PLoS One. 2022 Jun 24;17(6):e0270608. doi: 10.1371/journal.pone.0270608 (PMC9231803; doi:10.1371/journal.pone.0270608)
Supplement: S2 Table — (DOCX) [file pone.0270608.s002.docx]

Supplementary table 2 Features of patients with mSI-NETs grouped by primary tumor surgery before and after PSM

| Characteristics | Before PSM | | |  | After PSM | | |
| --- | --- | --- | --- | --- | --- | --- | --- |
|  | Non- primary tumor surgery | Primary tumor surgery | p |  | Non- primary tumor surgery | Primary tumor surgery | p |
| Insurance Recode |  |  | 0.131 |  |  |  | 0.155 |
| No/Unknown | 84(18.10%) | 224(15.17%) |  |  | 47(19.18%) | 60(24.49%) |  |
| Insured | 380(81.90%) | 1253(84.83%) |  |  | 198(80.82%) | 185(75.51%) |  |
| Marital status |  |  | 0.126 |  |  |  | 1.000 |
| Single/Unknown | 195(42.03%) | 562(38.05%) |  |  | 96(39.18%) | 96(39.18%) |  |
| Married | 269(57.97%) | 915(61.95%) |  |  | 149(60.82%) | 149(60.82%) |  |
| Race |  |  | 0.035 |  |  |  | 0.278 |
| Non-whites | 85(18.32%) | 211(14.29%) |  |  | 46(18.78%) | 37(15.10%) |  |
| White | 379(81.68%) | 1266(85.71%) |  |  | 199(81.22%) | 208(84.90%) |  |
| Age |  |  | 0.001 |  |  |  | 1.000 |
| <60 | 150(32.33%) | 60140.69%) |  |  | 84(34.29%) | 84(34.29%) |  |
| ≥60 | 314(67.67%) | 876(59.31%) |  |  | 161(65.71%) | 161(65.71%) |  |
| Sex |  |  | 0.107 |  |  |  | 0.416 |
| Female | 216(46.55%) | 751(50.85%) |  |  | 116(47.35%) | 125(51.02%) |  |
| Male | 248(53.45%) | 726(49.15%) |  |  | 129(52.65%) | 120(48.98%) |  |
| Primary tumor site |  |  | <0.001 |  |  |  | 0.170 |
| Duodenum | 86(18.53%) | 50(3.39%) |  |  | 43(17.55%) | 30(12.24%) |  |
| Jejunum and Ileum | 126(27.16%) | 962(65.13%) |  |  | 85(34.69%) | 100(40.82%) |  |
| Unknown | 252(54.31%) | 465(31.48%) |  |  | 117(47.76%) | 115(46.94%) |  |
| Grade |  |  | <0.001 |  |  |  | 1.000 |
| I | 127(27.37%) | 886(59.99%) |  |  | 88(35.92%) | 88(35.92%) |  |
| II | 36(7.76%) | 295(19.98%) |  |  | 28(11.43%) | 28(11.43%) |  |
| III/IV | 27(5.82%) | 56(3.78%) |  |  | 12(4.90%) | 12(4.90%) |  |
| Unknown | 274(59.05%) | 240(16.25%) |  |  | 117(47.75%) | 117(47.75%) |  |
| T stage |  |  | <0.001 |  |  |  | 0.272 |
| T1-2 | 66(14.22%) | 183(12.39%) |  |  | 57(23.27%) | 40(16.33%) |  |
| T3 | 48(10.34%) | 699(47.33%) |  |  | 35(14.29%) | 36(14.69%) |  |
| T4 | 39(8.41%) | 549(37.17%) |  |  | 25(10.20%) | 30(12.25%) |  |
| Unknown | 311(67.03%) | 46(3.11%) |  |  | 128(52.24%) | 139(56.73%) |  |
| N stage |  |  | <0.001 |  |  |  | 0.219 |
| N0 | 217(46.77%) | 282(19.09%) |  |  | 128(52.24%) | 111(45.31%) |  |
| N1-2 | 105(22.63%) | 1157(78.34%) |  |  | 55(22.45%) | 70(28.57%) |  |
| Unknown | 142(30.60%) | 38(2.57%) |  |  | 62(25.31%) | 64(26.12%) |  |
| Metastatic operation |  |  | <0.001 |  |  |  | 1.000 |
| No/unknown | 428(92.24%) | 855(57.89%) |  |  | 214(87.35%) | 214(87.35%) |  |
| Yes | 36(7.76%) | 622(42.11%) |  |  | 31(12.65%) | 31(12.65%) |  |
| Chemotherapy |  |  | <0.001 |  |  |  | 1.000 |
| No/Unknown | 359(77.37%) | 1252(84.77%) |  |  | 200(81.63%) | 200(81.63%) |  |
| Yes | 105(22.63%) | 225(15.23%) |  |  | 45(18.37%) | 45(18.37%) |  |
| Tumor size |  |  | <0.001 |  |  |  | 1.000 |
| <5cm | 137(29.53%) | 1249(84.56%) |  |  | 131(53.47%) | 131(53.47%) |  |
| ≥5cm | 25(5.39%) | 118(7.99%) |  |  | 21(8.57%) | 21(8.57%) |  |
| Unknown | 302(65.08%) | 110(7.45%) |  |  | 93(37.96%) | 93(37.96%) |  |
| Metastatic site |  |  | <0.001 |  |  |  | 0.570 |
| Liver | 257(55.39%) | 802(54.30%) |  |  | 125(51.02%) | 117(47.76%) |  |
| Lung | 28(6.03%) | 56(3.79%) |  |  | 15(6.12%) | 14(5.71%) |  |
| Brain and bone | 53(11.42%) | 64(4.33%) |  |  | 23(9.39%) | 18(7.35%) |  |
| Unknown | 126(27.16%) | 555(37.58%) |  |  | 82(33.47%) | 96(39.18%) |  |
