# Supplementary material for: Survival effects of primary and metastatic surgical treatment in metastatic small intestinal tumors: A propensity score–matching study
Source: PLoS One. 2022 Jun 24;17(6):e0270608. doi: 10.1371/journal.pone.0270608 (PMC9231803; doi:10.1371/journal.pone.0270608)
Supplement: S3 Table — (DOCX) [file pone.0270608.s003.docx]

Supplementary table 3 Features of patients with mSIA grouped by metastasis surgery before and after PSM

| Characteristics | Before PSM | | |  | After PSM | | |
| --- | --- | --- | --- | --- | --- | --- | --- |
|  | Non-surgery of metastasis | Surgery of metastasis | p |  | Non-surgery of metastasis | Surgery of metastasis | p |
| Insurance Recode |  |  | 0.617 |  |  |  | 0.842 |
| No/Unknown | 532(30.82%) | 118(32.15%) |  |  | 96(47.76%) | 98(48.76%) |  |
| Insured | 1194(69.18%) | 249(67.85%) |  |  | 105(52.24%) | 103(51.25%) |  |
| Marital status |  |  | 0.182 |  |  |  | 0.920 |
| Single/Unknown | 776(44.96%) | 151(41.14%%) |  |  | 85(42.29%) | 84(41.79%) |  |
| Married | 950(55.04%) | 216(58.86%) |  |  | 116(57.71%) | 117(58.21%) |  |
| Race |  |  | 0.725 |  |  |  | 0.826 |
| Non-whites | 491(28.45%) | 100(27.25%) |  |  | 59(29.35%) | 57(28.36%) |  |
| White | 1235(71.55%) | 267(72.75%) |  |  | 142(70.65%) | 144(71.64%) |  |
| Age |  |  | <0.001 |  |  |  | 0.914 |
| <60 | 468(27.11%) | 162(38.69%) |  |  | 63(31.34%) | 62(30.81%) |  |
| ≥60 | 1258(72.89%) | 205(61.31%) |  |  | 138(68.66%) | 139(69.19%) |  |
| Sex |  |  | 0.234 |  |  |  | 0.690 |
| Female | 797(46.18%) | 182(49.59%) |  |  | 94(46.74%) | 98(48.78%) |  |
| Male | 929(53.82%) | 185(50.41%) |  |  | 107(53.26%) | 103(51.22%) |  |
| Primary tumor site |  |  | <0.001 |  |  |  | 0.966 |
| Duodenum | 1080(62.57%) | 143(38.96%) |  |  | 93(46.23%) | 92(45.73%) |  |
| Jejunum and Ileum | 396(22.94%) | 137(37.33%) |  |  | 74(36.82%) | 73(36.34%) |  |
| Unknown | 250(14.49%) | 87(23.71%) |  |  | 34(16.95%) | 36(17.93%) |  |
| Grade |  |  | 0.093 |  |  |  | 0.968 |
| I | 73(4.23%) | 15(4.09%) |  |  | 4(1.99%) | 3(1.49%) |  |
| II | 567(32.85%) | 143(38.96%) |  |  | 88(43.78%) | 86(42.79%) |  |
| III/IV | 549(31.81%) | 115(31.34%) |  |  | 60(29.85%) | 60(29.85%) |  |
| Unknown | 537(31.11%) | 94(25.61%) |  |  | 49(24.38%) | 52(25.87%) |  |
| T stage |  |  | <0.001 |  |  |  | 0.974 |
| T1-2 | 242(14.02%) | 26(7.08%) |  |  | 10(4.98%) | 11(5.47%) |  |
| T3 | 271(15.71%) | 77(20.98%) |  |  | 39(19.40%) | 36(17.91%) |  |
| T4 | 648(37.54%) | 205(55.86%) |  |  | 109(54.23%) | 112(55.72%) |  |
| Unknown | 565(32.73%) | 59(16.08%) |  |  | 43(21.39%) | 42(20.90%) |  |
| N stage |  |  | <0.001 |  |  |  | 0.165 |
| N0 | 706(40.90%) | 131(35.69%) |  |  | 75(37.31%) | 63(31.34%) |  |
| N1-2 | 689(39.92%) | 192(52.32%) |  |  | 87(43.28%) | 106(52.74%) |  |
| Unknown | 331(19.18%) | 44(11.99%) |  |  | 39(19.41%) | 32(15.92%) |  |
| Primary tumor surgery |  |  | <0.001 |  |  |  | 1.000 |
| No/unknown | 1169(67.73%) | 114(31.06%) |  |  | 85(42.29%) | 85(42.29%) |  |
| Yes | 557(32.27%) | 253(68.94%) |  |  | 116(57.71%) | 116(57.71%) |  |
| Chemotherapy |  |  | 0.080 |  |  |  | 0.470 |
| No/Unknown | 706(40.90%) | 132(35.97%) |  |  | 78(38.81%) | 71(35.32%) |  |
| Yes | 1020(59.10%) | 235(64.03%) |  |  | 123(61.94%) | 130(64.68%) |  |
| Tumor size |  |  | <0.001 |  |  |  | 0.978 |
| <5cm | 544(31.52%) | 170(46.32%) |  |  | 97(48.26%) | 99(49.25%) |  |
| ≥5cm | 321(18.60%) | 78(21.25%) |  |  | 29(14.43%) | 28(13.93%) |  |
| Unknown | 861(49.88%) | 119(32.43%) |  |  | 75(37.31%) | 74(36.82%) |  |
| Metastatic site |  |  | <0.001 |  |  |  | 0.776 |
| Liver | 469(27.17%) | 49(13.35%) |  |  | 20(9.95%) | 21(10.45%) |  |
| Lung | 154(8.92%) | 43(11.72%) |  |  | 11(5.47%) | 16(7.96%) |  |
| Brain and bone | 139(8.06%) | 18(4.90%) |  |  | 7(3.48%) | 6(2.99%) |  |
| Unknown | 964(55.85%) | 257(70.03%) |  |  | 163(81.10%) | 158(78.60%) |  |
