# Supplementary material for: Survival effects of primary and metastatic surgical treatment in metastatic small intestinal tumors: A propensity score–matching study
Source: PLoS One. 2022 Jun 24;17(6):e0270608. doi: 10.1371/journal.pone.0270608 (PMC9231803; doi:10.1371/journal.pone.0270608)
Supplement: S4 Table — (DOCX) [file pone.0270608.s004.docx]

Supplementary table 4 Features of patients with mSI-NETs grouped by metastasis surgery before and after PSM

| Characteristics | Before PSM | | |  | After PSM | | |
| --- | --- | --- | --- | --- | --- | --- | --- |
|  | Non-surgery of metastasis | Surgery of metastasis | p |  | Non-surgery of metastasis | Surgery of metastasis | p |
| Insurance Recode |  |  | 0.103 |  |  |  | 0.502 |
| No/Unknown | 216(16.84%) | 92(13.98%) |  |  | 114(18.63%) | 105(17.16%) |  |
| Insured | 1067(83.16%) | 566(86.02%) |  |  | 498(81.37%) | 507(82.84%) |  |
| Marital status |  |  | 0.007 |  |  |  | 1.000 |
| Single/Unknown | 528(41.15%) | 229(34.80%) |  |  | 214(34.97%) | 214(34.97%) |  |
| Married | 755(58.85%) | 429(65.20%) |  |  | 398(65.03%) | 398(65.03%) |  |
| Race |  |  | 0.825 |  |  |  | 0.077 |
| Non-whites | 194(15.12%) | 102(15.50%) |  |  | 71(11.60%) | 92(15.03%) |  |
| White | 1089(84.88%) | 556(84.50%) |  |  | 541(88.40%) | 520(84.97%) |  |
| Age |  |  | <0.001 |  |  |  | 1.000 |
| <60 | 455(35.46%) | 296(44.98%) |  |  | 261(42.65%) | 261(42.65%) |  |
| ≥60 | 828(64.54%) | 362(55.02%) |  |  | 351(57.35%) | 351(57.35%) |  |
| Sex |  |  | 0.042 |  |  |  | 0.067 |
| Female | 618(48.17%) | 349(53.04%) |  |  | 292(47.71%) | 324(52.94%) |  |
| Male | 665(51.83%) | 309(46.96%) |  |  | 320(52.29%) | 288(47.06%) |  |
| Primary tumor site |  |  | <0.001 |  |  |  | 0.337 |
| Duodenum | 104(8.11%) | 32(4.86%) |  |  | 20(3.27%) | 30(4.90%) |  |
| Jejunum and Ileum | 665(51.83%) | 423(64.29%) |  |  | 399(65.20%) | 397(64.87%) |  |
| Unknown | 514(40.06%) | 203(30.85%) |  |  | 193(31.53%) | 185(30.23%) |  |
| Grade |  |  | <0.001 |  |  |  | 1.000 |
| I | 618(48.17%) | 395(60.03%) |  |  | 378(61.76%) | 378(61.76%) |  |
| II | 193(15.04%) | 138(20.97%) |  |  | 124(20.26%) | 124(20.26%) |  |
| III/IV | 65(5.07%) | 18(2.74%) |  |  | 16(2.62%) | 16(2.62%) |  |
| Unknown | 407(31.72%) | 107(16.26%) |  |  | 94(15.36%) | 94(15.36%) |  |
| T stage |  |  | <0.001 |  |  |  | 0.597 |
| T1-2 | 157(12.24%) | 92(13.98%) |  |  | 76(12.42%) | 89(14.54%) |  |
| T3 | 459(35.78%) | 288(43.77%) |  |  | 283(46.24%) | 264(43.14%) |  |
| T4 | 351(27.35%) | 237(36.02%) |  |  | 212(34.64%) | 220(35.95%) |  |
| Unknown | 316(24.63%) | 41(6.23%) |  |  | 41(6.70%) | 39(6.37%) |  |
| N stage |  |  | <0.001 |  |  |  | 0.565 |
| N0 | 364(28.37%) | 135(20.52%) |  |  | 134(21.90%) | 124(20.26%) |  |
| N1-2 | 763(59.47%) | 499(75.84%) |  |  | 451(73.69%) | 466(76.14%) |  |
| Unknown | 156(12.16%) | 24(3.64%) |  |  | 27(4.41%) | 22(3.58%) |  |
| Primary tumor surgery |  |  | <0.001 |  |  |  | 1.000 |
| No/unknown | 428(33.36%) | 36(5.47%) |  |  | 35(5.72%) | 35(5.72%) |  |
| Yes | 855(66.64%) | 622(94.53%) |  |  | 577(94.28%) | 577(94.28%) |  |
| Chemotherapy |  |  | 0.043 |  |  |  | 1.000 |
| No/Unknown | 1049(81.76%) | 562(85.41%) |  |  | 534(87.25%) | 534(87.25%) |  |
| Yes | 234(18.24%) | 96(14.59%) |  |  | 78(12.75%) | 78(12.75%) |  |
| Tumor size |  |  | <0.001 |  |  |  | 1.000 |
| <5cm | 837(65.24%) | 549(83.43%) |  |  | 517(84.48%) | 517(84.48%) |  |
| ≥5cm | 100(7.79%) | 43(6.53%) |  |  | 36(5.88%) | 36(5.88%) |  |
| Unknown | 346(26.97%) | 66(10.04%) |  |  | 59(9.64%) | 59(9.64%) |  |
| Metastatic site |  |  | 0.001 |  |  |  | 0.176 |
| Liver | 659(51.36%) | 400(60.79%) |  |  | 278(45.42%) | 267(43.63%) |  |
| Lung | 65(5.07%) | 19(2.89%) |  |  | 15(2.45%) | 16(2.61%) |  |
| Brain and bone | 83(6.47%) | 34(5.17%) |  |  | 14(2.29%) | 28(4.58%) |  |
| Unknown | 476(37.10%) | 205(31.15%) |  |  | 305(49.84%) | 301(49.18%) |  |
