# Supplementary material for: Survival effects of primary and metastatic surgical treatment in metastatic small intestinal tumors: A propensity score–matching study
Source: PLoS One. 2022 Jun 24;17(6):e0270608. doi: 10.1371/journal.pone.0270608 (PMC9231803; doi:10.1371/journal.pone.0270608)
Supplement: S5 Table — (DOCX) [file pone.0270608.s005.docx]

Supplementary table 5 Features of patients with mSIA grouped by primary surgical approach before and after PSM

| Characteristics | Before PSM | | |  | After PSM | | |
| --- | --- | --- | --- | --- | --- | --- | --- |
|  | Local surgery | Intestine-ectomy | p |  | Local surgery | Intestine-ectomy | p |
| Insurance Recode |  |  | 0.767 |  |  |  | 0.913 |
| No/Unknown | 156(33.40%) | 118(34.40%) |  |  | 72(41.62%) | 71(41.04%) |  |
| Insured | 311(66.60%) | 225(65.60%) |  |  | 101(58.38%) | 102(58.96%) |  |
| Marital status |  |  | 0.009 |  |  |  | 0.277 |
| Single/Unknown | 182(38.97%) | 165(48.10%) |  |  | 69(39.88%) | 79(45.66%) |  |
| Married | 285(61.03%) | 178(51.90%) |  |  | 104(60.12%) | 94(54.34%) |  |
| Race |  |  | 0.007 |  |  |  | 0.191 |
| Non-whites | 109(23.34%) | 109(31.77%) |  |  | 44(25.43%) | 55(31.79%) |  |
| White | 358(76.66%) | 234(68.23%) |  |  | 129(74.57%) | 118(68.21%) |  |
| Age |  |  | 0.122 |  |  |  | 0.826 |
| <60 | 179(38.33%) | 150(43.73%) |  |  | 70(40.46%) | 68(39.31%) |  |
| ≥60 | 288(61.67%) | 193(56.27%) |  |  | 103(59.54%) | 105(60.69%) |  |
| Sex |  |  | <0.001 |  |  |  | 0.745 |
| Female | 190(40.69%) | 185(53.94%) |  |  | 73(42.20%) | 76(43.93%) |  |
| Male | 277(59.31%) | 158(46.06%) |  |  | 100(57.80%) | 97(56.07%) |  |
| Primary tumor site |  |  | <0.001 |  |  |  | 0.345 |
| Duodenum | 78(16.70%) | 93(27.11%) |  |  | 30(17.34%) | 39(22.54%) |  |
| Jejunum and Ileum | 250(53.53%) | 176(51.32%) |  |  | 96(55.49%) | 96(55.49%) |  |
| Unknown | 139(29.77%) | 74(21.57%) |  |  | 47(27.17%) | 38(21.97%) |  |
| Grade |  |  | 0.482 |  |  |  | 0.983 |
| I | 20(4.28%) | 15(4.37%) |  |  | 6(3.47%) | 5(2.89%) |  |
| II | 212(45.40%) | 137(39.94%) |  |  | 78(45.09%) | 79(45.66%) |  |
| III/IV | 191(40.90%) | 156(45.48%) |  |  | 80(46.24%) | 81(46.82%) |  |
| Unknown | 44(9.42%) | 35(10.21%) |  |  | 9(5.20%) | 8(4.63%) |  |
| T stage |  |  | 0.214 |  |  |  | 0.852 |
| T1-2 | 25(5.35%) | 11(3.21%) |  |  | 4(2.31%) | 2(1.16%) |  |
| T3 | 154(32.98%) | 99(28.86%) |  |  | 49(28.33%) | 50(28.90%) |  |
| T4 | 263(56.32%) | 215(62.68%) |  |  | 116(67.05%) | 116(67.05%) |  |
| Unknown | 25(5.35%) | 18(5.25%) |  |  | 4(2.31%) | 5(2.89%) |  |
| N stage |  |  | 0.128 |  |  |  | 1.000 |
| N0 | 165(35.33%) | 108(31.49%) |  |  | 51(29.48%) | 51(29.48%) |  |
| N1-2 | 272(58.24%) | 221 (64.43%) |  |  | 121(69.94%) | 121(69.94%) |  |
| Unknown | 30(6.43%) | 14(4.08%) |  |  | 1(0.58%) | 1(0.58%) |  |
| Metastatic operation |  |  | 0.130 |  |  |  | 0.910 |
| No/unknown | 331(70.88%) | 226(65.89%) |  |  | 114(65.90%) | 115(66.47%) |  |
| Yes | 136(29.12%) | 117(34.11%) |  |  | 59(34.10%) | 58(33.53%) |  |
| Chemotherapy |  |  | 0.121 |  |  |  | 0.819 |
| No/Unknown | 194(41.54%) | 124(36.15%) |  |  | 56(32.37%) | 58(33.53%) |  |
| Yes | 273(58.46%) | 219(63.85%) |  |  | 117(67.63%) | 115(66.47%) |  |
| Tumor size |  |  | 0.867 |  |  |  | 0.984 |
| <5cm | 266(56.96%) | 193(56.27%) |  |  | 112(64.74%) | 111(64.16%) |  |
| ≥5cm | 118(25.27%) | 92(26.82%) |  |  | 43(24.86%) | 43(24.86%) |  |
| Unknown | 83(17.77%) | 58(16.91%) |  |  | 18(10.40%) | 19(10.98%) |  |
| Metastatic site |  |  | 0.343 |  |  |  | 0.560 |
| Liver | 76(16.27%) | 47(13.71%) |  |  | 22(12.72%) | 22(12.72%) |  |
| Lung | 38(8.14%) | 29(8.45%) |  |  | 9(5.20%) | 8(4.62%) |  |
| Brain and bone | 28(6.00%) | 13(3.79%) |  |  | 0(0.00%) | 2(1.16%) |  |
| Unknown | 325(69.59%) | 254(74.05%) |  |  | 142(82.08%) | 141(81.50%) |  |
