# Supplementary material for: Survival effects of primary and metastatic surgical treatment in metastatic small intestinal tumors: A propensity score–matching study
Source: PLoS One. 2022 Jun 24;17(6):e0270608. doi: 10.1371/journal.pone.0270608 (PMC9231803; doi:10.1371/journal.pone.0270608)
Supplement: S6 Table — (DOCX) [file pone.0270608.s006.docx]

Supplementary table 6 Features of patients with mSI-NETs grouped by primary surgical approach before and after PSM.

| Characteristics | Before PSM | | |  | After PSM | | |
| --- | --- | --- | --- | --- | --- | --- | --- |
|  | Local surgery | Intestine-ectomy | p |  | Local surgery | Intestine-ectomy | p |
| Insurance Recode |  |  | 0.512 |  |  |  | 0.607 |
| No/Unknown | 122(14.63%) | 102(15.86%) |  |  | 77(14.31%) | 83(15.43%) |  |
| Insured | 712(85.37%) | 541(84.14%) |  |  | 461(85.69%) | 455(84.57%) |  |
| Marital status |  |  | 0.943 |  |  |  | 0.755 |
| Single/Unknown | 318(38.13%) | 244(37.95%) |  |  | 212(39.41%) | 207(38.48%) |  |
| Married | 516(61.87%) | 399(62.05%) |  |  | 326(60.59%) | 331(61.52%) |  |
| Race |  |  | 0.239 |  |  |  | 0.215 |
| Non-whites | 127(15.23%) | 84(13.06%) |  |  | 81(15.06%) | 67(12.45%) |  |
| White | 707(84.77%) | 559(86.94%) |  |  | 457(84.94%) | 471(87.55%) |  |
| Age |  |  | 0.022 |  |  |  | 1.000 |
| <60 | 318(38.13%) | 283(44.01%) |  |  | 233(43.31%) | 233(43.31%) |  |
| ≥60 | 516(61.87%) | 360(55.99%) |  |  | 305(56.69%) | 305(56.69%) |  |
| Sex |  |  | 0.092 |  |  |  | 0.127 |
| Female | 408(48.92%) | 343(53.34%) |  |  | 254(47.21%) | 279(51.86%) |  |
| Male | 426(51.08%) | 300(46.66%) |  |  | 284(52.79%) | 259(48.14%) |  |
| Primary tumor site |  |  | <0.001 |  |  |  | 0.976 |
| Duodenum | 21(2.52%) | 29(4.51%) |  |  | 12(2.23%) | 13(2.42%) |  |
| Jejunum and Ileum | 503(60.31%) | 459(71.38%) |  |  | 335(62.27%) | 333(61.90%) |  |
| Unknown | 310(37.17%) | 155(24.11%) |  |  | 191(35.50%) | 192(35.69%) |  |
| Grade |  |  | 0.974 |  |  |  | 1.000 |
| I | 500(59.95%) | 386(60.03%) |  |  | 343(63.75%) | 343(63.75%) |  |
| II | 168(20.14%) | 127(19.75%) |  |  | 106(19.71%) | 106(19.71%) |  |
| III/IV | 30(3.60%) | 26(4.05%) |  |  | 16(2.97%) | 16(2.97%) |  |
| Unknown | 136(16.31%) | 104(16.17%) |  |  | 73(13.57%) | 73(13.57%) |  |
| T stage |  |  | 0.448 |  |  |  | 0.638 |
| T1-2 | 109(13.07%) | 74(11.51%) |  |  | 74(13.75%) | 63(11.71%) |  |
| T3 | 398(47.72%) | 301(46.81%) |  |  | 256(47.58%) | 264(49.07%) |  |
| T4 | 298(35.73%) | 251(39.04%) |  |  | 199(36.99%) | 205(38.10%) |  |
| Unknown | 29(3.48%) | 17(2.64%) |  |  | 9(1.68%) | 6(1.12%) |  |
| N stage |  |  | 0.003 |  |  |  | 1.000 |
| N0 | 185(22.18%) | 97(15.09%) |  |  | 75(13.94%) | 75(13.94%) |  |
| N1-2 | 628(75.30%) | 529 (82.27%) |  |  | 461(85.69%) | 461(85.69%) |  |
| Unknown | 21(2.52%) | 17(2.64%) |  |  | 2(0.37%) | 2(0.37%) |  |
| Metastatic operation |  |  | 0.277 |  |  |  | 0.806 |
| No/unknown | 493(59.11%) | 362(56.30%) |  |  | 305(56.69%) | 301(55.95%) |  |
| Yes | 341(40.89%) | 281(43.70%) |  |  | 233(43.31%) | 237(44.05%) |  |
| Chemotherapy |  |  | 0.186 |  |  |  | 1.000 |
| No/Unknown | 716(85.85%) | 536(83.36%) |  |  | 480(89.22%) | 480(89.22%) |  |
| Yes | 118(14.15%) | 107(16.64%) |  |  | 58(10.78%) | 58(10.78%) |  |
| Tumor size |  |  | 0.120 |  |  |  | 1.000 |
| <5cm | 715(85.73%) | 534(83.05%) |  |  | 485(90.15%) | 485(90.15%) |  |
| ≥5cm | 56(6.72%) | 62(9.64%) |  |  | 32(5.95%) | 32(5.95%) |  |
| Unknown | 63(7.55%) | 47(7.31%) |  |  | 21(3.90%) | 21(3.90%) |  |
| Metastatic site |  |  | 0.321 |  |  |  | 1.000 |
| Liver | 450(53.95%) | 352(54.83%) |  |  | 318(59.11%) | 318(59.11%) |  |
| Lung | 35(4.20%) | 21(3.27%) |  |  | 16(2.97%) | 16(2.97%) |  |
| Brain and bone | 30(3.60%) | 34(5.30%) |  |  | 17(3.16%) | 17(3.16%) |  |
| Unknown | 319(38.25%) | 235(36.60%) |  |  | 187(34.76%) | 187(34.76%) |  |
