# Supplementary material for: Survival effects of primary and metastatic surgical treatment in metastatic small intestinal tumors: A propensity score–matching study
Source: PLoS One. 2022 Jun 24;17(6):e0270608. doi: 10.1371/journal.pone.0270608 (PMC9231803; doi:10.1371/journal.pone.0270608)
Supplement: S7 Table — (DOCX) [file pone.0270608.s007.docx]

Supplementary table 7 Features of patients with mSIA grouped by RNE before and after PSM

| Characteristics | Before PSM | | |  | After PSM | | |
| --- | --- | --- | --- | --- | --- | --- | --- |
|  | RNE<8 | RNE≥8 | p |  | RNE<8 | RNE≥8 | p |
| Insurance Recode |  |  | <0.001 |  |  |  | 0.159 |
| No/Unknown | 178(40.00%) | 83(25.30%) |  |  | 62(50.41%) | 51(41.46%) |  |
| Insured | 267(60.00%) | 245(74.70%) |  |  | 61(49.59%) | 72(58.54%) |  |
| Marital status |  |  | 0.295 |  |  |  | 0.517 |
| Single/Unknown | 184(41.35%) | 148(45.12%) |  |  | 53(43.09%) | 48(39.02%) |  |
| Married | 261(58.65%) | 180(54.88%) |  |  | 70(56.91%) | 75(60.98%) |  |
| Race |  |  | 0.884 |  |  |  | 0.492 |
| Non-whites | 120(26.97%) | 90(27.44%) |  |  | 41(33.33%) | 36(29.27%) |  |
| White | 325(73.03%) | 238(72.56%) |  |  | 82(66.67%) | 87(70.73%) |  |
| Age |  |  | 0.886 |  |  |  | 1.000 |
| <60 | 180(40.45%) | 131(39.94%) |  |  | 44(35.77%) | 44(35.77%) |  |
| ≥60 | 265(59.55%) | 197(60.06%) |  |  | 79(64.23%) | 79(64.23%) |  |
| Sex |  |  | 0.808 |  |  |  | 0.898 |
| Female | 205(46.07%) | 154(46.95%) |  |  | 56(45.53%) | 57(46.34%) |  |
| Male | 240(53.93%) | 174(53.05%) |  |  | 67(54.47%) | 66(53.66%) |  |
| Primary tumor site |  |  | <0.001 |  |  |  | 0.348 |
| Duodenum | 98(22.02%) | 61(18.60%) |  |  | 28(22.76%) | 20(16.26%) |  |
| Jejunum and Ileum | 208(46.74%) | 202(61.59%) |  |  | 66(53.66%) | 76(61.79%) |  |
| Unknown | 139(31.24%) | 65(19.81%) |  |  | 29(23.58%) | 27(21.95%) |  |
| Grade |  |  | 0.001 |  |  |  | 1.000 |
| I | 23(5.17%) | 11(3.35%) |  |  | 2(1.63%) | 2(1.63%) |  |
| II | 199(44.72%) | 138(42.07%) |  |  | 55(44.72%) | 55(44.72%) |  |
| III/IV | 170(38.20%) | 161(49.09%) |  |  | 61(49.59%) | 61(49.59%) |  |
| Unknown | 53(11.91%) | 18(5.49%) |  |  | 5(4.06%) | 5(4.06%) |  |
| T stage |  |  | <0.001 |  |  |  | 1.000 |
| T1-2 | 25(5.62%) | 7(2.13%) |  |  | 2(1.63%) | 2(1.63%) |  |
| T3 | 146(32.81%) | 98(29.88%) |  |  | 41(33.33%) | 41(33.33%) |  |
| T4 | 236(53.03%) | 221(67.38%) |  |  | 79(64.23%) | 79(64.23%) |  |
| Unknown | 38(8.54%) | 2(0.61%) |  |  | 1(0.81%) | 1(0.81%) |  |
| N stage |  |  | <0.001 |  |  |  | 1.000 |
| N0 | 216(48.54%) | 48(14.63%) |  |  | 30(24.39%) | 30(24.39%) |  |
| N1-2 | 193(43.37%) | 278(84.76%) |  |  | 92(74.80%) | 92(74.80%) |  |
| Unknown | 36(8.09%) | 2(0.61%) |  |  | 1(0.81%) | 1(0.81%) |  |
| Primary tumor surgery |  |  | <0.001 |  |  |  | 1.000 |
| Localized surgery | 287(64.49%) | 157(47.87%) |  |  | 74(60.16%) | 74(60.16%) |  |
| Intestine-ectomy | 158(35.51%) | 171(52.13%) |  |  | 49(39.84%) | 49(39.84%) |  |
| Metastatic operation |  |  | 0.542 |  |  |  | 0.565 |
| No/unknown | 309(69.43%) | 221(67.38%) |  |  | 92(74.80%) | 88(71.54%) |  |
| Yes | 136(30.54%) | 107(32.62%) |  |  | 31(25.20%) | 35(28.46%) |  |
| Chemotherapy |  |  | 0.002 |  |  |  | 1.000 |
| No/Unknown | 195(43.82%) | 107(32.62%) |  |  | 51(41.46%) | 51(41.46%) |  |
| Yes | 250(56.18%) | 221(67.38%) |  |  | 72(58.54%) | 72(58.54%) |  |
| Tumor size |  |  | <0.001 |  |  |  | 1.000 |
| <5cm | 257(57.75%) | 190(57.93%) |  |  | 82(66.67%) | 82(66.67%) |  |
| ≥5cm | 85(19.10%) | 114(34.76%) |  |  | 30(24.39%) | 30(24.39%) |  |
| Unknown | 103(23.15%) | 24(7.31%) |  |  | 11(8.94%) | 11(8.94%) |  |
| Metastatic site |  |  | 0.150 |  |  |  | 1.000 |
| Liver | 57(12.81%) | 61(18.60%) |  |  | 11(8.94%) | 11(8.94%) |  |
| Lung | 36(8.09%) | 28(8.54%) |  |  | 6(4.88%) | 6(4.88%) |  |
| Brain and bone | 22(4.94%) | 17(5.18%) |  |  | 5(4.07%) | 5(4.07%) |  |
| Unknown | 330(74.16%) | 222(67.68%) |  |  | 101(82.11%) | 101(82.11%) |  |
