# Supplementary material for: Survival effects of primary and metastatic surgical treatment in metastatic small intestinal tumors: A propensity score–matching study
Source: PLoS One. 2022 Jun 24;17(6):e0270608. doi: 10.1371/journal.pone.0270608 (PMC9231803; doi:10.1371/journal.pone.0270608)
Supplement: S8 Table — (DOCX) [file pone.0270608.s008.docx]

Supplementary table 8 Features of patients with mSI-NETs grouped by RNE before and after PSM

| Characteristics | Before PSM | | |  | After PSM | | |
| --- | --- | --- | --- | --- | --- | --- | --- |
|  | RNE<8 | RNE≥8 | p |  | RNE<8 | RNE≥8 | p |
| Insurance Recode |  |  | 0.004 |  |  |  | 0.160 |
| No/Unknown | 105(18.32%) | 108(12.77%) |  |  | 57(17.17%) | 44(13.25%) |  |
| Insured | 468(81.68%) | 738(87.23%) |  |  | 275(82.83%) | 288(86.75%) |  |
| Marital status |  |  | 0.537 |  |  |  | 1.000 |
| Single/Unknown | 224(39.09%) | 317(37.47%) |  |  | 128(38.55%) | 128(38.55%) |  |
| Married | 349(60.91%) | 529(62.53%) |  |  | 204(61.45%) | 204(61.45%) |  |
| Race |  |  | 0.001 |  |  |  | 0.692 |
| Non-whites | 101(17.63%) | 98(11.58%) |  |  | 61(18.37%) | 65(19.58%) |  |
| White | 472(82.37%) | 478(88.42%) |  |  | 271(81.63%) | 267(80.42%) |  |
| Age |  |  | 0.017 |  |  |  | 1.000 |
| <60 | 213(37.17%) | 368(43.50%) |  |  | 129(38.86%) | 129(38.86%) |  |
| ≥60 | 360(62.83%) | 478(56.50%) |  |  | 203(61.14%) | 203(61.14%) |  |
| Sex |  |  | 0.262 |  |  |  | 0.535 |
| Female | 282(49.21%) | 442(52.25%) |  |  | 163(49.10%) | 171(51.51%) |  |
| Male | 291(50.79%) | 404(47.75%) |  |  | 169(50.90%) | 161(48.49%) |  |
| Primary tumor site |  |  | <0.001 |  |  |  | 0.568 |
| Duodenum | 36(6.28%) | 14(1.65%) |  |  | 14(4.22%) | 9(2.71%) |  |
| Jejunum and Ileum | 305(53.23%) | 620(73.29%) |  |  | 187(56.33%) | 189(56.93%) |  |
| Unknown | 232(40.49%) | 212(25.06%) |  |  | 131(39.45%) | 134(40.36%) |  |
| Grade |  |  | 0.017 |  |  |  | 1.000 |
| I | 335(58.46%) | 528(62.41%) |  |  | 220(66.27%) | 220(66.27%) |  |
| II | 105(18.33%) | 178(21.04%) |  |  | 62(18.67%) | 62(18.67%) |  |
| III/IV | 25(4.36%) | 29(3.43%) |  |  | 4(1.20%) | 4(1.20%) |  |
| Unknown | 108(18.85%) | 111(13.12%) |  |  | 46(13.86%) | 46(13.86%) |  |
| T stage |  |  | <0.001 |  |  |  | 1.000 |
| T1-2 | 83(14.49%) | 95(11.23%) |  |  | 44(13.25%) | 44(13.25%) |  |
| T3 | 294(51.31%) | 382(45.15%) |  |  | 173(52.11%) | 173(52.11%) |  |
| T4 | 169(29.49%) | 359(42.44%) |  |  | 114(34.34%) | 114(34.34%) |  |
| Unknown | 27(4.71%) | 10(1.18%) |  |  | 1(0.30%) | 1(0.30%) |  |
| N stage |  |  | <0.001 |  |  |  | 1.000 |
| N0 | 218(38.05%) | 62(7.33%) |  |  | 42(12.65%) | 42(12.65%) |  |
| N1-2 | 332(57.94%) | 779(92.08%) |  |  | 290(87.35%) | 290(87.35%) |  |
| Unknown | 23(4.01%) | 5(0.59%) |  |  | 0(0.00%) | 0(0.00%) |  |
| Primary tumor surgery |  |  | <0.001 |  |  |  | 0.485 |
| Localized surgery | 374(65.27%) | 426(50.36%) |  |  | 172(51.81%) | 163(49.10%) |  |
| Intestine-ectomy | 199(34.73%) | 420(49.64%) |  |  | 160(48.19%) | 169(50.90%) |  |
| Metastatic operation |  |  | 0.103 |  |  |  | 0.579 |
| No/unknown | 344(60.03%) | 471(55.67%) |  |  | 204(61.45%) | 197(59.34%) |  |
| Yes | 229(39.97%) | 375(44.33%) |  |  | 128(38.55%) | 135(40.66%) |  |
| Chemotherapy |  |  | 0.217 |  |  |  | 1.000 |
| No/Unknown | 478(83.42%) | 726(85.82%) |  |  | 293(88.25%) | 293(88.25%) |  |
| Yes | 95(16.58%) | 120(14.18%) |  |  | 39(11.75%) | 39(11.75%) |  |
| Tumor size |  |  | 0.009 |  |  |  | 1.000 |
| <5cm | 476(83.07%) | 739(87.35%) |  |  | 312(93.98%) | 312(93.98%) |  |
| ≥5cm | 47(8.20%) | 67(7.92%) |  |  | 15(4.52%) | 15(4.52%) |  |
| Unknown | 50(8.73%) | 40(4.73%) |  |  | 5(1.50%) | 5(1.50%) |  |
| Metastatic site |  |  | 0.064 |  |  |  | 0.539 |
| Liver | 291(50.79%) | 486(57.45%) |  |  | 171(51.51%) | 181(54.52%) |  |
| Lung | 24(4.19%) | 32(3.78%) |  |  | 10(3.01%) | 5(1.51%) |  |
| Brain and bone | 23(4.01%) | 38(4.49%) |  |  | 12(3.61%) | 10(3.01%) |  |
| Unknown | 235(41.01%) | 290(34.28%) |  |  | 139(41.87%) | 136(40.96%) |  |
